# Optimizing genotype quality metrics for individual exomes and cohort analysis

**DOI:** 10.1186/1753-6561-6-S6-P42

**Published:** 2012-10-01

**Authors:** Paul MK Gordon, Leo Dimnik, Ryan Lamont, Micheil Innes, Francois Bernier, Jillian Parboosingh

**Affiliations:** 1 Alberta Children's Hospital Research Institute (ACHRI) Genomics Platform, University of Calgary, Calgary, Alberta, Canada; 2 Genetic Laboratory Services, Alberta Health Services, Calgary, Alberta, Canada; 3 Department of Medical Genetics, University of Calgary, Calgary, Alberta, Canada

## Background

Few evidence-based best practice bioinformatics guidelines exist for genotyping using next-generation sequencing data, especially colorspace data produced by Life Technologies sequencers. Dozens of software packages can perform the various steps required, and genome features such as pseudogenes or large paralogous gene families are problematic. High false positive and negative rates can compound the difficulty of cohort analysis.

## Materials and methods

Using a Sanger-validated set of 32 *BRCA* gene regions from 16 patients, high-throughput colorspace (Life Technologies) sequencing performance was optimized by comparing various combinations of sequence aligners, re-aligners, de-duplicators, quality re-calibrators and genotype callers. Independently, six exomes were captured using the Agilent SureSelect v3 kit. The optimized pipeline was applied, and results were compared to microarray genotyping to characterize false positives and negatives. A further four exomes were pair-end sequenced on both the Life Technologies 5500x1 and Illumina HiSeq sequencers to check platform concordance. Variant metrics for each exome were compared to the literature.

In the clinic, individual exomes are manually triaged by a medical geneticist, and salient variants are confirmed by Sanger sequencing. For disease cohorts, software was developed to isolate variants possibly causing monogenic rare diseases, taking likely false positives into account.

## Results

Using results from Life Technologies' reference genome aligner, the intersection of single nucleotide polymorphism (SNP) calls from FreeBayes [1] (with SamTools [2] de-duplication) and Life Technologies' diBayes (with Picard de-duplication) was optimal. Using reads realigned by the Broad Institute Genome Analysis Toolkit (GATK) [3], the intersection of insertion and deletion calls from FreeBayes and Atlas2 [4] was optimal. A threshold of 14% variant reads for true heterozygous calls was observed.

For bases with 10× coverage, variant calls are on average 98.9% concordant with SNP microarrays (versus 99.2% microarray technical reproducibility [5]). False positive and negative variant rates are each approximately 0.5%, with all false positives called heterozygous. Concordance with Illumina variant calls from a standard GATK pipeline was 95.2%. GATK produced more novel variants, especially in non-unique genomic regions: such variants are flagged with caveats in the colorspace pipeline. In a dominant heterozygous model analysis of five Nager syndrome patients, our cohort analysis software excluded 15 of 19 candidate genes, based mainly on a preponderance of genotype caveats.

Many published metrics for SNP quality control are based on a small number of genomes elucidated using other technologies, but Table 1 shows overall agreement with the optimized colorspace pipeline results.

**Table 1:** Quality metrics reported in the literature, and the optimized colorspace genotyping results.

	Ideal	Colorspace exome average
Protein coding	0.048% [6]	0.052
Non-coding	>coding	0.056
Non-synonymous	45% [7]	46.2
Homozygous	37-40% [8]	38.7
Coding SNP transitions:transversions	2.8-3.0:1 [9]	3.2:1
Non-coding SNP transitions:transversions	2.0-2.2:1 [9]	2.3:1
CDS novel (versus dbSNP135)	N/A	0.58

N/A, not applicable

## Conclusions

Low false positive and negative rates using colorspace data can be achieved by: first, reporting only concurrent variants from ultiple methods; and second, reporting caveats where the reference sequence is not unique. Accurate calls and caveats enable major cohort gene triage when modeling diseases caused by monogenic rare variants.
